# Reliability of measurement of active trunk movement in wheelchair basketball players

**DOI:** 10.1371/journal.pone.0225515

**Published:** 2019-11-21

**Authors:** Jolanta Marszałek, Bartosz Molik

**Affiliations:** Department of Rehabilitation, Jozef Pilsudski University of Physical Education in Warsaw, Poland; University of Memphis, UNITED STATES

## Abstract

The study aim was to assess the reliability to active trunk movements measurement in four sitting positions in wheelchair basketball players and to check their trunk movements in these positions. Eighteen volunteer wheelchair basketball athletes, with a minimum of five years’ training experience, were asked to perform the maximum range of active trunk movement in three planes in four sitting positions (in a sports wheelchair with straps, without straps, on a table with feet on the floor, on a table without foot support). The range of movement was measured by the Kinect for Windows V2 sensor twice (with one-week interval). To assess the reliability, different statistical methods were used for each movement: significance of differences between the results (*p*-value), interclass correlation coefficient (ICC) and minimal detectable change (MDC). The limits of agreement analysis (LOA) were calculated. Differences between trunk movements in four positions were checked by the MANOVA (Wilk’s Lambda and ETA^2^ were calculated if data were normally distributed). The significance level was set at α < .05. Friedman ANOVA and non-parametric Wilcoxon test with the Bonferroni correction were applied when data were not normally distributed. The significance level after Bonferroni correction was set at α < .013 (α = p/k, where p = .05, k–number of positions = 4). The measurement of active trunk movement in each plane was reliable (*p* > .05, no differences between the results, “very good”ICC, between .96-.99). In the position with straps, the trunk movement was significantly bigger than in other positions (*p* < .05), except for the position without straps (*p* > .05). The Kinect for Windows V2 sensor measured active trunk movement in a reliable manner and it can be recommended as a reliable tool for measuring trunk function. Utilizing straps by wheelchair basketball players increases their trunk movement.

## Introduction

Trunk function significantly influences the quality of movements in a chair in everyday activities of wheelchair users such as sitting, propelling a wheelchair, transferring, eating, dressing up/undressing, etc. [1–7]. Many factors affect trunk function e.g. trunk range of movement, postural control or muscle activation after the center of mass displacement [8]. Trunk movement assessment is one of the main points to evaluate in the classification process in wheelchair sports, because sports activities are depended on trunk movement (flexion, extension, lateral flexion, rotation) in three planes: the sagittal plane (forward), the coronal plane (sideways) and the transverse plane (vertical). According to their trunk abilities, athletes are divided into different sports classes in many parasports like wheelchair rugby [9–13], wheelchair basketball [13–18], wheelchair fencing [19], wheelchair track [20], paraskiing [21–23] or wheelchair tennis. Therefore, the examination of validity and reliability of methods used for assessing trunk function is important, needed and supported by the International Paralympic Committee as a part of classification systems development; i.e., conducting research according to evidence-based classification approach in each parasport classification discipline to search a gold standard method to assess and divide players into classes [24].

There are several methods of assessing trunk function in a clinical way; e.g., the Trunk Impairment Classification system (TIC) used in wheelchair rugby classification [10, 11] or the Trunk Impairment Scale (TIS) used for assessing trunk function of people with multiple sclerosis in many sports [25]. The American Spinal Injury Association (ASIA) impairment scale (AIS) helps to assess the medical level of athletes’ trunk function in paraskiing [21, 26]. The Test-Table-Test Board performed in a special stand [21, 26], special motorized plate on which a sit-ski can be placed to test athletes’ trunk stability [23] or the unique sensorized frame with mounted electrical-drive sledge to test trunk control are helpful in assessing trunk function in specific settings in paraskiing athletes. In general, balance tests (static and dynamic) [6, 9, 12, 16], variations of reach tests [14, 27], trunk inclination tests using force plates [28] and maximal static trunk strength measurement [5, 16, 17, 20] are used for trunk assessment. However, according to evidence-based classification approach, there is a need to find appropriate methods, tools or tests related to movement specificity in a given sport that would support or change the current type of trunk assessment [29]. For example, the functional observation of active trunk movement in players in terms of the volume of trunk action during a wheelchair basketball game serves as a basis for dividing athletes into sports classes [30]. The importance of active trunk movement for wheelchair basketball players' classification was checked and confirmed by Vanlandewijck et al. [13], and is considered as one of the main points in global athletes’ evaluation in this sport discipline. The volume of trunk action is assessed by classifiers in three planes, i.e. the sagittal plane (forward), the coronal plane (sideways) and the transverse plane (vertical) based on the classification rules in real surroundings (players’ observations on the court in a sports wheelchair with straps) [30]. It would be helpful if there was a tool which could support classifiers or could confirm their observations, and it would be interesting to indicate in which sitting position the volume of action of wheelchair basketball player is the biggest (sitting position in a wheelchair with or without straps or sitting position on a table). Currently, there are no studies concerning which would be helpful for wheelchair basketball classifiers.

In a literature review, a study was found in which wheelchair users’ trunk movement was video recorded [3]. Curtis et al. [3] analyzed and measured sitting trunk mobility in wheelchair users focusing on the differences of trunk stabilization due to types of belts, but they did not discuss the methods which they used. The idea of a video analysis of wheelchair basketball players’ trunk movement could be helpful and easy to use for classifiers. We found that current, new and easy methods of assessing human body movements in all joints are being discovered, e.g. the Kinect sensor–used specifically to support the rehabilitation process [31–34]. This tool (small hardware and easy software) would make it easy to present and to assess active trunk functions in wheelchair athletes even though some authors noticed that the results of the range of movement could vary and they commented on this aspect in their studies [31, 32]. Bonnechere et al. [31] found good reliability of the Kinect sensor (*p* > .05) and significant differences (*p* < .05) in the range of movement between two measurement tools: the Kinect sensor and stereophotogrammetry systems in hip abduction, knee flexion, shoulder abduction, elbow flexion [31]. Huber et al. [32] found good reliability in shoulder range of movement and some discrepancies in shoulder rotation [32]. Therefore, examining the full potential of the Kinect sensor to support the measurement (assessment) of trunk function seems to be necessary.

Based on the above needs and ideas, the aim of this study was to assess the reliability of the measurement of active trunk movement in four different sitting positions in wheelchair basketball players and to check their trunk movements related to these four sitting positions.

## Materials and methods

### Participants

Eighteen wheelchair basketball players from the United States of America (USA) university league, participated in this study (nine men and nine women; mean body height = 1.7±.1 [m], mean body mass = 66.3±.1 [kg], and mean body height = 1.6±.3 [m], mean body mass = 55.3±.4 [kg], respectively). They had been playing wheelchair basketball for at least five years.

The health conditions of the study participants were as follows: spinal cord injury (n = 5), spina bifida (n = 4), cerebral palsy (n = 2), lower limb amputation (n = 1), transverse myelitis (n = 2) and other (one person with Brittle Bone Disease of a lower limb, one person after lower limb bone sarcoma, one person with Charcot-Marie-Tooth disease type 2a, one person with joint immobility; n = 4). Seven players received between 1.0 and 2.5 classification points, while 11 players received between 3.0 and 4.5 classification points according to the International Wheelchair Basketball Federation (IWBF) manual of classification [25]. Athletes were classified by a panel of classifiers (minimum two classifiers, national and international level classifiers from the USA).

Participants had five practice sessions on the court per week (one practice—two hours) and 3 hours of lifting strength training per week. The exclusion criteria were the lack of the class according to the classification system, little experience in playing wheelchair basketball (less than five years) and no possibility of sitting on a table without upper limbs support. The health condition, the sport class and experience in playing wheelchair basketball were determined based on the interview.

All athletes were informed about the study and signed a consent form. The Institutional Review Board (IRB; The Senate Commission of Science Research Ethics at Jozef Pilsudski University of Physical Education in Warsaw) approved this study (SKE 01-54/2017). All the procedures of this study were completed in accordance with the ethical standards as described in the Declaration of Helsinki.

### Protocol

Participants were asked to perform six trunk movements, three repetition each: trunk extension, trunk flexion, trunk right lateral flexion, trunk left lateral flexion, trunk right rotation, trunk left rotation. The movements were performed in four different positions: sitting in a sport wheelchair with athletes’ straps (W+S), sitting in a sport wheelchair without straps (W-S), sitting on a table with feet set on the floor (T+F), sitting on a table without foot support (T-F). These four positions were chosen to check differences in trunk movements in different body positions (sitting in a sport wheelchair or on a table, with feet set on the floor or without feet set on the floor). The resting periods between each of the six trunk movements were minimum 60 seconds long. The resting periods between the repetitions in each trunk movement were 15 seconds long. The trunk inclination in the sport wheelchair (W+S and W-S) was calculated as the individual’s wheelchair angle between the seat and the backrest. The trunk inclination on a table (T+F and T-F) was calculated as the angle between the trunk and the thighs while the athlete was seated on a table and no straps were used. The athletes were instructed to perform each movement with upper limbs kept naturally bent at the elbows (90 degrees), with a neutral position of the shoulders, with their maximal engagement in each movement until they knew that the movement was active and performed without compensation. All athletes prepared to participate in this study themselves (five minutes of warm up, checking their active limits in each movement). One week later the protocol was repeated. Between measurements, the athletes continued their practices and strength training.

The maximal voluntary range of trunk movement (in degrees) for each movement (active movement) was measured by the Kinect for Windows V2 sensor (The Kinetisense module: Functional 3D Range-of-Motion) and calculated by the Windows Software Development Kit (SDK). This is a low-cost, portable device which does not require any sensors to be attached to the body device and can measure the range of movement in each joint in non-laboratory surroundings. The Kinetisense application that captures pictures and videos of human movement is compatible with the Kinect for Windows V2 sensor and the SDK and is necessary to conduct measurements. The Kinect V2 sensor (Functional 3D Range-of-Motion) was placed on a tripod at a height of 1.5 m and at a distance of 1.80–2.0 m (depending on a sitting body height of a participant). The sensor was placed facing the sagittal plane (extension and flexion; the participant was placed sideways to the camera) as well as the coronal and the transverse plane (for lateral flexions and rotations, respectively; the participant was placed facing the camera) according to the manual of measurements with the use of the Kinect V2 sensor [35]. This device (hardware) is made up of RGB video camera, depth sensor and multi-array microphone. The SDK that includes sensor streams, skeletal tracking and advanced audio capabilities, was used [36].

### Statistical analysis

A mean of three repetitions of each trunk movement (trunk extension, trunk flexion, trunk right lateral flexion, trunk left lateral flexion, trunk right rotation, trunk left rotation) was taken into account in the analyses. All calculations and analyses were performed using the SPSS IBM Statistics 24 for Windows (IBM Corp., Armonk, NY, USA). The analysis of the quantitative data was based on arithmetic means and standard deviations (*SD*). The difference between the results from the first day of test (Results 1) and the results from the second day of test (Results 2) (subtraction) were counted. Discrepancies of Results 1 and Results 2 were assessed using limits of agreement analysis (LOA) with Bland Altman plots [37]. The Kolmogorov-Smirnov test was applied in order to examine the distribution of results.

To assess the reliability of the measurements, Results 1 (the results from the first measurement) and Results 2 (the results from the second measurement) in the tests were compared with the use of the t-test in the case data showed normal distribution and the non-parametric Wilcoxon test in the case data did not show normal distribution. The level of significance was set at α < .05. The second step to assess reliability was to determine the relationship between the first measurement and the second measurement. Additionally, the interclass correlation coefficient (ICC) were calculated to determine the relative test-retest reliability of the results of active trunk movement obtained with the Kinect V2 sensor. The ICC values were defined as ‘poor’ for values below .20, ‘fair’ for values between .21 and .40, ‘moderate’ for values between .41 and .60, ‘good’ for values between .61 and .80, and ‘very good’ for values between .81 and 1.0 [38]. The standard error of measurement (SEM) and the minimal detectable change at the 90% confidence interval (MDC_90_) were calculated to establish the absolute reliability [39]. The SEM was calculated according to the following formula:

$$SEM=SD\sqrt{\left(1-r\right)}$$ (1),

where *SD* is the standard deviation of the measurement and *r* is the reliability coefficient (test-retest reliability in the form of the ICC for the study group). The SEM was multiplied by the square root of the number of measurements [40]. The MDC was calculated from the formula:

$$MDC90=1.65\;x\;SEM\;x\sqrt{2}$$ (2)

The MDC values are being increasingly used when interpreting results and determining whether a change between repeated tests is a random variation or a true change in performance [39, 41]. The MDC is the magnitude of change that a measurement must demonstrate to exceed the anticipated measurement error and variability. If a change in a score that is greater than MDC_90_ occurs in either direction, one can be 90% confident that the difference does not stem from the measurement error or patient variability [42]. %MDC_90_ is the MDC_90_ expressed as a percentage of the measurement mean.

To assess the differences in trunk range of motion between the four sitting positions and the three planes, a multivariate analysis of variance test (MANOVA; full factorial for dependent variables and repeated measurements test), Wilk’s Lambda (λ) and ETA^2^ (effect size) were calculated if data showed normal distribution. The level of significance was set at α < .05. Friedman ANOVA and non-parametric Wilcoxon test with the Bonferroni correction were applied in the case data did not show normal distribution. The level of significance after Bonferroni correction was set at α < .013 (α = p/k, where p = .05, k–number of passions (4 positions).

## Results

Table 1 shows all the results of trunk range of movement [in degrees] from the first day of test (Result 1) and the second day of test (Result 2) for four sitting positions and the three planes.

**Table 1:** Mean, standard deviation, and reliability results for trunk movements in the three planes and four sitting positions (n = 18).

I	II	III	IV	V	VI	VII	VIII	IX	X	XI
Starting position	Trunk movement	Result 1 [degrees]	Result 2 [degrees]	Result 1 - Result 2 [degrees]	LOA	p	ICC	SEM	MDC_90_	%MDC_90_
		Mean1±SD1	Mean2±SD2							
W+S	Extension	31.8±17.6	30.5±16.1	1.4±1.5	[-1.63 4.35]	.35	.99	1.8	4.1	13
	Flexion	55.6±22.6	^53.9±21.3	1.6±1.3	[-.98 4.20]	^.21	.99	2.4	5.5	10
	Left Flexion	36.1±18.3	37.0±18.1	-1.0±.2	[-2.03 .04]	.06	.99	1.2	2.7	7
	Right Flexion	36.6±18.9	37.0±19.2	-.4±-.3	[-1.31 .53]	.39	.99	.6	1.4	4
	Left Rotation	32.7±14.6	33.2±14.8	-.5±-.2	[-1.89 .92]	.48	.98	2.3	5.4	16
	Right Rotation	33.5±14.3	33.2±14.1	.3±.3	[-1.12 1.67]	.69	.99	1.8	4.1	12
W-S	Extension	24.9±14.9	23.7±14.9	1.2±.0	[-1.17 3.49]	.31	.99	.8	1.9	8
	Flexion	^45.4±27.0	^43.6±28.5	1.8±-1.5	[-.16 2.67]	^.11	.99	.9	2.0	4
	Left Flexion	24.5±13.6	22.4±13.7	2.1±-.1	[.59 3.54]	.06	.99	.9	2.0	8
	Right Flexion	22.5±12.1	21.0±12.6	1.5±-.5	[-1.05 4.10]	.23	.98	1.6	3.8	17
	Left Rotation	28.3±12.6	28.3±12.8	.0±-.2	[-1.26 1.35]	.94	.99	1.0	2.8	8
	Right Rotation	31.4±16.4	30.8±15.4	.6±1.0	[-.66 1.84]	.33	.99	1.3	2.9	9
T+F	Extension	13.5±9.2	^13.1±8.8	.4±.4	[-.73 1.49]	.79	.99	.7	1.7	12
	Flexion	37.6±26.5	37.0±25.9	.5±.6	[-.60 1.66]	.61	.99	1.7	3.9	10
	Left Flexion	^12.3±7.0	12.0±6.7	.3±.3	[-.83 1.40]	^.92	.99	.4	.9	7
	Right Flexion	12.0±6.4	11.6±6.4	.4±-.1	[-.98 1.81]	.79	.98	.9	2.1	18
	Left Rotation	27.6±16.0	27.8±17.3	-.2±1.4	[-1.62 1.25]	.57	.99	1.8	4.1	15
	Right Rotation	28.3±15.7	27.1±16.4	1.1±-.8	[-.35 2.63]	.25	.99	.7	1.6	6
T-F	Extension	12.9±8.8	12.6±7.6	.3±1.3	[-.40 1.23]	.16	.99	.8	1.8	14
	Flexion	23.7±17.5	23.4±17.5	.3±.1	[-.90 1.47]	.62	.99	1.0	2.2	9
	Left Flexion	10.5±5.3	11.1±5.2	-.6±.1	[-1.39 .24]	.16	.98	.7	1.7	16
	Right Flexion	11.9±5.8	10.9±5.4	1.0±.3	[-.01 1.98]	.05	.98	.8	1.7	15
	Left Rotation	24.5±12.1	25.3±12.0	-.7±.1	[-1.70 .27]	.14	.98	1.8	4.1	17
	Right Rotation	24.4±11.5	24.2±11.5	.2±.0	[-.66 1.03]	.66	.99	.6	1.5	6

p < .05; Result 1—Result 2 –the difference between Results 1 and Results 2; LOA—limits of agreements (Bland and Altman); p–p-value; ICC–Interclass Correlation Coefficient; SEM–standard error of measurement; MDC–minimal detected change; %MDC_90_ –MDC_90_ expressed as a percent of measurement mean; SD–standard deviation; W+S–wheelchair with straps; W-S–wheelchair without straps; T+F–sitting on a table with feet on the floor; T-F–sitting on a table without foot support

^ - data that did not show normal distribution and the non-parametric Wilcoxon test/the Spearman's rank correlation coefficient were applied

Results for the reliability are reported in Table 1 in columns VII (*p-*values), and VIII (ICC). There were no significant differences between Results 1 and Results 2 (VII; *p* > .05). Relative reliability (VIII) was “very good” for each measurement (ICC > .81). The difference between the results from the first day of test (Results 1) and the results from the second day of test (Results 2) was introduced in column V. The 95% LOA Bland and Altman between the measurements were not more than 3° in all trunk movements in all measured positions, except for the extension W+S (5.98°), flexion W+S (5.18°), right flexion W-S (5.15°), and extension W-S (4.66°; column VI).

Results for absolute reliability are reported in Table 1 in columns VIII (SEM), IX (MDC90), and X (%MDC). All mean differences between Result 1 and Result 2 in the W+S, W-S, T+F and T-F position (except data of Left Flexion in W-S position that was bigger than MDC_90_) were smaller than MDC_90_ (Table 1). Each statistical analysis (*p*-value, ICC, SEM and MDC_90_) confirmed that the trunk measurements in each position and for each direction are reliable.

In Table 2, the differences of active trunk range of movement in the four sitting positions and three planes are reported. Active trunk movements in the sagittal plane (flexion) in the W+S position were significantly different (the range of movement was greater; *p* < .05) compared to the T+F and T-F positions and the movements in the coronal plane (lateral flexion) in the W+S position were significantly different (the range of movement was greater; *p* < .05) compared to the W-S, T+F and T-F positions. Active trunk flexion was significantly larger in the T+F position compared to T-F (*p* < .05), while active trunk extension and movements in the coronal plane and the transverse plane did not show statistical differences (*p* > .05). Movements in the transverse plane did not show statistical differences in the W+S and W-S, as well as in the T+F and T-F positions (*p* > .05). Multivariate analysis of variance test (MANOVA; full factorial for dependent variables and repeated measurements test) was calculated and Wilk’s Lambda (λ) were introduced for extension as λ = .21; F(3,14) = 17.7; p < .001; ETA^2^ = .79, left flexion as λ = .27; F(3,14) = 12.9; p < .001; ETA^2^ = .73, right flexion as λ = .28; F(3,14) = 12.2; p < .001; ETA^2^ = .72, left rotation as λ = .54; F(3,14) = 4.0; p < 0.03; ETA^2^ = .46, right rotation as λ = .56; F(3,14) = 3.7; p < .04; ETA^2^ = .44. The Bonferroni adjustment in post-hoc test of multiple comparisons was used.

**Table 2:** Differences in the results of active trunk movement measurements in four different sitting positions in wheelchair athletes.

Starting position	Trunk movement	W-S		T+F		T-F	
		Difference [degrees]	p	Difference [degrees]	p	Difference [degrees]	p
W+S	Extension	6.9	.341	17.2	.001	17.6	.001
	Flexion	10.0	^.039	17.5	^.001	31.2	^.001
	Left Flexion	12.8	.001	23.4	.001	24.7	.001
	Right Flexion	14.6	.001	24.3	.001	24.8	.001
	Left Rotation	4.6	.141	6.1	.130	9.0	.012
	Right Rotation	2.3	1.0	6.0	.213	9.2	.025
W-S	Extension			10.4	.001	10.8	.003
	Flexion			7.5	^.001	21.2	^.001
	Left Flexion			10.6	.001	11.9	.001
	Right Flexion			9.7	.005	10.0	.006
	Left Rotation			1.5	1.0	4.6	.138
	Right Rotation			3.8	1.0	7.1	.301
T+F	Extension					.4	1.0
	Flexion					13.7	^.001
	Left Flexion					1.3	.30
	Right Flexion					.5	1.0
	Left Rotation					3.0	.560
	Right Rotation					3.3	.363

p < .05; W+S–wheelchair with straps; W-S–wheelchair without straps; T+F–sitting on a table with feet on the floor; T-F–sitting on a table without foot support

^—data that did not show normal distribution and Friedman ANOVA with the Bonferroni correction was applied (the level of significance after Bonferroni correction was set at α < .013)

## Discussion

The main aim of this study was to assess the reliability of the measurement of active trunk movement in the three planes in four different sitting positions in wheelchair athletes performed with the Kinect V2 sensor. The second aim was to check the participants’ trunk movements related to these four sitting positions. The biggest achievement of this study is that the reliability of the measurement of active trunk movement that was performed with the use of the Kinect V2 sensor [35] in the sagittal plane, the coronal plane and the transverse plane is confirmed. Different statistical methods were applied to assess the reliability of the measurements, i.e. significance of differences between the results (*p*-value), the interclass correlation coefficient (ICC) and minimal detectable change (MDC). The measurement in all the applied positions, i.e. in a sitting position in a wheelchair with straps (W+S) and without straps (W-S) as well as in a sitting position on a table with feet on the floor (T+F) and without foot support (T-F), showed overall good reliability (no significant differences between measurements in test-retest, i.e. *p* > .05, “very good” ICC, i.e. ICC > .81, low MDC_90_, i.e. MDC_90_ between .0 and 5.5, and low %MDC_90_, i.e. MDC_90_ between 4 and 18). This information is necessary to allow conducting future research using this device to assess trunk movement in a wheelchair and/or on a table.

Despite no studies have been found regarding the reliability of trunk movement measured by the Kinect V2 sensor, few studies have investigated the range of movement of the shoulder, elbow, hips and knees in healthy able-bodied participants (non-sport participants) [31, 32]. In particular, Bonnechere et al. [31] and Huber et al. [32] found moderate to good reliability of the range of movement in the above-mentioned joints (ICC > .7). Apart from several some positive aspects of the Kinect V2 sensor; e.g., the fact that it is a like low-cost, portable and easy-to-use device that can measure the range of movement in each joint in non-laboratory surroundings (e.g. a patience’s house), these authors mentioned that there were found some inconsistencies such as different results in the range of movement in the same joint measured by the Kinect V2 sensor and another device chosen to assess joint movement [31, 32].

Comparing the trunk movements between the four tested positions pointed out some significant differences. The first two positions (the position in a wheelchair with straps and the position in a wheelchair without straps) were chosen to assess active trunk movement since we sought to investigate whether there is a difference between active trunk movement in a wheelchair which is specially adapted to a player (where straps are used for stability during a game) and active trunk movement without being strapped. It was confirmed that active trunk movement in six directions is significantly larger when a player uses straps (like in a game), and probably they feel safer, more stable and more confident in a wheelchair with straps to move the trunk further in all directions (straps support individual’s impaired muscles in each movement). This idea can be supported by Horak’s et al. [8] research and reflections on muscle responses throughout the body because of displacements of the total-body center of mass (COM) from equilibrium [8].

Curtis et al. [3] also assessed the influence of using belts (chest and tight belts) in wheelchair basketball players on their active trunk movement (functional reach movement) in the sagittal plane and the transverse plane [3]. They revealed partially similar results and conclusions as in the current study, i.e. wheelchair basketball players representing each class benefited from use of belts in their functional active trunk movement in flexion and rotation. In the current study, it was observed that belts will not be helpful in the rotation in the transverse plane. No significant differences were noticed between active trunk rotation in W+S and W-S. It is suspected that the trunk in this movement does not move beyond the support area in a sitting position, and the belt is not so helpful as in the case of flexion. In our study, displacement of the COM was not observed. However, in Horak’s et al. [8] study is underlined that because of displacements of the COM from equilibrium, muscle have to respond quickly throughout the body to keep the COM in equilibrium. It seems that in rotation the COM is not displayed as much and straps do not have to help muscles in trunk rotation to achieve bigger movement in transverse plane.

The two positions on a table, i.e. T+F and T-F, were helpful in understanding how the lower limbs’ support helps to achieve a greater range of movement in flexion (only this range of movement was significantly greater in the T+F position compared to T-F). The current results mean that lower limbs (their setting on the floor) significantly support and improve the volume of trunk action in flexion. During trunk flexion in a sitting position, the COM is moving forward [8]. The presence of lower limbs on the floor/footrest (no lower limbs amputations or foot setting on the floor/footrest) prevents the COM to move beyond the base and has influence on better trunk stabilization (significantly bigger trunk flexion) [8]. Proper settings of a wheelchair like the inclination angle of the backrest to the seat or the seat and the footrest distance related to anthropometrical length of shanks, can help to achieve better trunk stabilization (trunk function) in the case lower limbs are amputated, characterized by weak muscle power or are no active (e.g. no muscle power of lower limbs in individuals with complete spinal cord injury people) [13].

Active trunk movement assessment in wheelchair basketball players performed with the use of the Kinect V2 sensor may be helpful for classifiers in trunk assessment in evidence-based classification process in the future. This measurement can be compared to classifiers’ assessment and can confirm or reject their final decision or be a solution in the assessment in a special situation, e.g. when classifiers have different opinions about the same player, they can include the Kinect V2 sensor in voluntary testing to assess trunk movements. Moreover, classifiers could assess players’ active trunk movement when players sit in their own wheelchair. Results from this study confirmed reliability of trunk measurement assessment when players are in their own sport wheelchair. This point is important because it is necessary to take into account sports-specific assessment and players’ adaptations, like wheelchair settings, in evidence-based classification assessment [43]. Probably, this device and software could also be used in different sports like wheelchair racing, wheelchair rugby, wheelchair tennis and other sports where active trunk movement is important when it comes to dividing athletes into different classes. On the other hand, this device could be used as supporting equipment in clinical practice on patients who are wheelchair users and by companies that help to adapt wheelchairs to players' needs and abilities as well as by coaches to improve players’ active trunk movement (the volume of trunk action). Having this system of measurement, would make it easier and faster to evaluate the position in which an individual has bigger trunk range of movement (bigger than this before the assessment and before the implication of new wheelchair settings) because their wheelchair settings like straps or the inclination angle between the seat and the backrest are better adapted to individual’s functional abilities.

### Study limitations and recommendations for future studies

The general idea of the current study is based on the participants' honesty. In each test, all the players were moving according to their abilities and they did not hide their capabilities. The results did not influence their classification points. It is not uncommon that many players want to be in a lower class than they are, and even though this device is reliable, it cannot prevent future athletes from cheating during the classification process, which was reported by Molik et al. [44] on the basis of athletes' opinions [44]. However, the Kinect V2 sensor could be utilized by a company to help wheelchair athletes to adapt sports wheelchairs appropriately to players’ abilities to achieve maximal possible active trunk movement (the maximal possible volume of trunk action).

In the future studies, the sample size should be larger in order to produce stronger evidence for the appropriateness of the current classification system and to confirm that the classifiers’ observation is a sufficient measure to divide players into different sports classes in wheelchair basketball. The next important point for future studies is the validity of the trunk movement measurement and accuracy of the measurement. That is why we recommend to continue investigation of the Kinect for Windows V2 sensor in trunk movement in standing and in sitting positions.

## Conclusions

The reliability of the measurement of active trunk movement in four different sitting positions in wheelchair athletes assessed with the Kinect V2 sensor was confirmed. Based on three types of statistical methods, it was concluded that this active trunk movement assessment of wheelchair basketball players performed with the use of the Kinect V2 sensor [35] is reliable and may support classifiers work in evidence-based classification process.

Utilizing straps in a wheelchair sitting position increases trunk movement of wheelchair basketball players.

## Supporting information

S1 File Results for PlosONE. (PDF)
