# Supplementary material for: Reliability of measurement of active trunk movement in wheelchair basketball players
Source: PLoS One. 2019 Nov 21;14(11):e0225515. doi: 10.1371/journal.pone.0225515 (PMC6872154; doi:10.1371/journal.pone.0225515)
Supplement: S1 File — (PDF) [file pone.0225515.s001.pdf]

No.	ex_wpluss	fl_wpluss	fllef_wpluss	flrig_wpluss	rotlef_wpluss	rotrig_wpluss	ex_wminuss	fl_wminuss
1	17,86	69,52	34,63	32,45	25,91	27,77	12,97	27,78
2	4,51	16,96	8,04	6,61	19,23	19,12	7,55	9,90
3	31,02	65,97	43,92	42,95	31,74	33,48	29,51	62,81
4	25,05	25,04	26,18	21,40	30,86	28,92	19,65	9,96
5	24,44	64,00	24,12	27,69	49,21	36,43	23,73	70,76
6	41,14	69,08	52,40	47,94	18,00	25,10	33,39	20,37
7	8,76	16,46	11,35	8,44	11,87	6,99	7,06	9,23
8	46,09	55,14	43,51	41,36	40,90	58,50	14,33	55,34
9	29,20	58,77	47,93	60,56	60,09	50,85	53,47	64,20
10	32,87	52,19	41,89	37,04	40,40	35,25	40,35	55,71
11	68,13	79,01	53,84	63,60	49,87	48,28	36,48	81,46
12	51,41	83,84	70,86	68,79	53,19	49,59	38,79	66,90
13	9,25	14,55	4,47	8,70	9,22	10,25	1,10	5,13
14	18,08	55,63	23,17	26,62	18,65	29,99	8,39	11,75
15	45,80	65,41	49,50	52,07	42,03	50,50	42,55	67,18
16	34,47	55,15	22,56	20,76	26,81	23,68	31,02	54,84
17	26,35	73,15	49,60	43,98	29,55	33,76	14,78	62,07
18	46,08	66,34	49,18	50,59	34,78	31,28	21,80	70,57

fllef_wminuss	flrig_wminuss	rotlef_wminuss	rotrig_wminuss	ex_bplusf	fl_bplusf	fllef_bplusf	flrig_bplusf	rotlef_bplusf
13,00	22,52	24,67	23,15	8,13	24,29	7,79	10,83	24,47
7,10	7,82	14,48	26,26	2,30	4,46	5,74	4,33	6,32
14,78	15,03	30,40	31,21	10,67	49,87	7,63	8,79	23,52
10,93	10,41	20,32	7,36	9,72	6,66	7,99	10,27	23,89
23,93	14,97	27,92	36,80	21,54	56,70	13,35	15,22	13,06
34,94	25,18	13,78	23,83					
10,73	8,02	7,39	7,02	5,26	12,39	8,50	9,74	8,05
26,99	25,23	33,74	58,55	11,18	49,27	15,07	13,02	24,83
37,47	41,23	46,77	40,99	28,75	55,97	19,80	23,86	54,19
35,58	38,42	39,49	41,37	19,30	18,03	20,10	10,55	31,56
38,88	27,83	49,67	53,35	29,11	81,37	25,17	19,66	51,83
53,48	46,13	40,75	34,57	24,90	57,57	17,73	18,33	45,14
3,68	5,10	9,31	7,67	2,55	1,37	2,75	1,37	3,80
7,68	10,23	25,92	32,43	4,66	3,65	6,23	7,52	19,19
28,48	24,35	37,63	57,16	16,80	64,09	16,70	12,04	49,81
21,67	14,60	35,29	28,14	12,45	39,32	9,91	12,50	30,37
30,67	31,81	17,06	15,90	7,58	50,74	6,84	5,63	23,62
21,93	21,94	34,81	33,29	11,39	58,10	15,54	17,12	37,16

rotrig_bplusf	ex_bminusf	fl_bminusf	fllef_bminusf	flrig_bminusf	rotlef_bminusf	rotrig_bminusf
24,34	12,46	22,06	9,47	11,65	18,16	20,02
9,88	2,53	3,88	6,38	7,06	7,94	6,86
17,26	14,09	9,99	7,41	8,86	14,18	11,44
18,48	6,56	7,30	8,62	9,40	18,02	24,06
21,68	25,89	42,34	14,85	15,10	21,45	22,11
	9,81	8,86	9,63	12,26	31,60	21,55
9,19	4,82	9,09	5,94	4,57	5,92	10,21
27,02	12,33	31,89	12,24	15,07	17,25	25,47
51,65	24,67	43,18	22,23	23,53	38,79	41,91
17,95	19,18	14,22	14,20	6,42	27,57	18,07
52,69	26,81	52,87	18,53	20,79	45,72	40,53
31,24	22,07	49,61	15,56	15,23	34,73	16,17
6,23	2,08	1,32	2,94	2,32	5,43	8,96
24,90	5,18	3,07	6,28	7,76	30,56	34,47
54,20	12,25	28,73	13,66	12,50	39,94	44,15
32,36	11,11	21,13	9,34	13,38	25,88	27,10
30,69	8,17	46,34	3,68	5,81	32,31	32,43
40,83	10,08	28,27	13,05	13,34	32,80	31,13
